# Peroral Endoscopic Myotomy (POEM) and Laparoscopic Heller Myotomy with Dor Fundoplication for Esophagogastric Junction Outflow Obstruction (EGJOO): a Comparison of Outcomes and Impact on Physiology

**DOI:** 10.1007/s11605-023-05844-0

**Published:** 2023-10-17

**Authors:** Inanc S. Sarici, Sven Eriksson, Mohamad Rassoul Abu-Nuwar, Jacob Kuzy, Margaret Gardner, Ping Zheng, Blair Jobe, Shahin Ayazi

**Affiliations:** 1 Foregut Division, Surgical Institute, Allegheny Health Network, 4815 Liberty Avenue, Suite 439, Pittsburgh, PA 15224 USA; 2 Chevalier Jackson Research Fellowship, Esophageal Institute, Western Pennsylvania Hospital, Pittsburgh, PA USA; 3 Department of Surgery, Drexel University, Philadelphia, PA USA

**Keywords:** Esophagogastric junction outflow obstruction (EGJOO), Lower esophageal sphincter (LES), Heller myotomy, Per oral endoscopic myotomy (POEM), GERD

## Abstract

**Introduction:**

Esophagogastric junction outflow obstruction (EGJOO) is an esophageal motility disorder characterized by failure of lower esophageal sphincter (LES) relaxation with preserved peristalsis. Studies have shown that Heller myotomy with Dor fundoplication (HMD) and per oral endoscopic myotomy (POEM) are effective treatments for EGJOO. However, there is paucity of data comparing the efficacy and impact of these two procedures. Therefore, the aim of this study was to compare outcomes and impact on esophageal physiology in patients undergoing HMD or POEM for primary EGJOO.

**Methods:**

This was a retrospective review of patients who underwent either HMD or POEM for primary EGJOO at our institution between 2013 and 2021. Favorable outcome was defined as an Eckardt score ≤ 3 at 1 year after surgery. GERD–HRQL questionnaire, endoscopy, pH monitoring, and high-resolution manometry (HRM) results at baseline and 1 year after surgery were compared pre- and post-surgery and between groups. Objective GERD was defined as DeMeester score > 14.7 or LA grade C/D esophagitis.

**Results:**

The final study population consisted of 52 patients who underwent HMD (*n* = 35) or POEM (*n* = 17) for EGJOO. At a mean (SD) follow-up of 24.6 (15.3) months, favorable outcome was achieved by 30 (85.7%) patients after HMD and 14 (82.4%) after POEM (*p* = 0.753). After HMD, there was a decrease GERD-HRQL total score (31 (22–45) to 4 (0–19); *p* < 0.001), and objective reflux (54.2 to 25.9%; *p* = 0.033). On manometry, there was a decrease in LES resting pressure (48 (34–59) to 13 (8–17); *p* < 0.001) and IRP (22 (17–28) to 8 (3–11); *p* < 0.001), but esophageal body characteristics did not change (*p* > 0.05). Incomplete bolus clearance improved (70% (10–90) to 10% (0–40); *p* = 0.010). After POEM, there was no change in the GERD-HRQL total score (*p* = 0.854), but objective reflux significantly increased (0 to 62%; *p* < 0.001). On manometry, there was a decrease in LES resting pressure (43 (30–68) to 31 (5–34); *p* = 0.042) and IRP (23 (18–33) to 12 (10–32); *p* = 0.048), DCI (1920 (1600–5500) to 0 (0–814); *p* = 0.035), with increased failed swallows (0% (0–30) to 100% (10–100); *p* = 0.032). Bolus clearance did not improve (*p* = 0.539). Compared to HMD, POEM had a longer esophageal myotomy length (11 (7–15)-vs-5 (5–6); *p* = 0.001), more objective reflux (*p* = 0.041), lower DCI (0 (0–814)-vs-1695 (929–3101); *p* = 0.004), and intact swallows (90 (70–100)-vs-0 (0–40); *p* = 0.006), but more failed swallows (100 (10–100); *p* = 0.018) and incomplete bolus clearance (90 (90–100)-vs-10 (0–40); *p* = 0.004).

**Conclusion:**

Peroral endoscopic myotomy and Heller myotomy with Dor fundoplication are equally effective at relieving EGJOO symptoms. However, POEM causes worse reflux and near complete loss of esophageal body function.

**Graphical Abstract:**

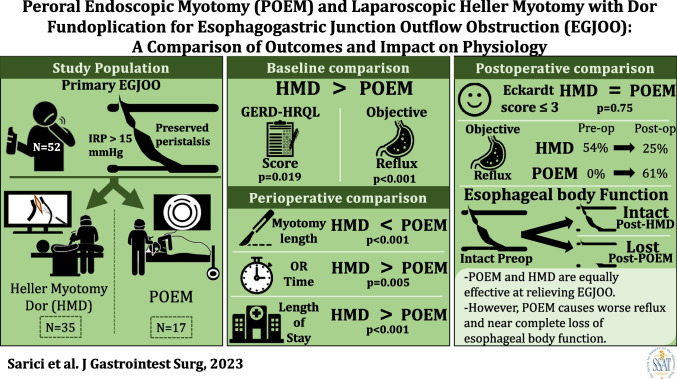

## Introduction

Esophagogastric junction outflow obstruction (EGJOO) is an esophageal motility disorder characterized by impaired relaxation of the lower esophageal sphincter (LES) with preserved peristalsis of the esophageal body.^[1]^ The etiology of EGJOO can be primary or secondary and is found in 3–24% of patients who undergo high resolution manometry (HRM).^[2, 3]^ Secondary EGJOO can be due to structural, postsurgical or a malignant process, and is best managed by addressing the underlying pathology.^[4]^ Conversely, primary EGJOO is an idiopathic disease process and is mainly managed through symptom palliation. Patients with mild symptoms may respond to pharmacotherapy, pneumatic dilation, or botulinum toxin injection.^[4]^ However, patients with refractory or severe symptoms may require surgical myotomy.^[5, 6]^

The goal of surgical myotomy is to disrupt the muscle fibers responsible for the hypertensive LES, thereby decreasing resistance and relieving symptoms. This can be done via a transabdominal approach with the Heller myotomy or an endoscopic approach known as peroral endoscopic myotomy (POEM). The advantage of the transabdominal approach is that it grants access to the hiatus, allowing hiatal hernia repair if needed, and can be performed in conjunction with a partial fundoplication to assist with reflux control after myotomy. The endoscopic approach eliminates the risks of laparoscopic surgery and enables the surgeon to extend the myotomy more proximally on to the esophageal body muscles so that myotomy length can be tailored to diseased segment length. Limited studies have demonstrated that both Heller myotomy and POEM can effectively palliate dysphagia in patients with EGJOO with success rates of 96 and 94%, respectfully.^[6, 7]^

Heller myotomy with Dor fundoplication (HMD) and POEM are well-established surgical procedures for esophageal motility disorders such as achalasia and its subtypes, and many studies have shown the safety and efficacy of both procedures.^[8, 9]^ However, studies of HMD and POEM for the management of EGJOO are limited with small sample size, short-term follow-up, and lack of complete postoperative objective data.^[5, 6]^ Additionally, no studies have compared the efficacy of these two surgical procedures. Therefore, this study aims to compare the outcomes and impact on esophageal physiology between HMD and POEM in patients with primary EGJOO.

## Materials and Methods

### Study Population

This was a retrospective review of prospectively collected data of patients who underwent either HMD or POEM due to primary EGJOO at Allegheny Health Network hospitals (Pittsburgh, PA) between 2013 and 2021. This study was evaluated and approved by the Institutional Review Board of the Allegheny Health Network (IRB Number 2021–239). Patients with a diagnosis of primary EGJOO who were 18 years or older and had at least 1-year follow-up after surgery were included in this study. Patients diagnosed with secondary EGJOO (e.g., hiatal hernia, stricture), achalasia, jackhammer esophagus, diffuse esophageal spasm, and other esophageal motility disorders were not included in this study.

### Disease-Related Quality of Life Measures

All patients were asked to complete validated questionnaires preoperatively and 1-year postoperatively, including Eckardt symptom score and Gastroesophageal Reflux Disease-Health-Related Quality of Life (GERD-HRQL). The Eckardt score was used to grade the severity of esophageal motility disorders and assess four symptoms: weight loss, dysphagia, retrosternal pain, and regurgitation. Each symptom was scored from 0 to 3 with an aggregate score between 0 and 12. A total Eckardt score greater than three was considered abnormal. The total GERD-HRQL was calculated by summing 16 disease specific symptom severity questions, ranging from 0 to 5. This questionnaire also assessed patient satisfaction and use of anti-secretory medications.

### Preoperative Clinical and Objective Evaluation

All patients underwent a comprehensive clinical evaluation focusing on their foregut symptoms. In addition, the routine preoperative objective assessment included the following tests:

#### Videoesophagram

Gross esophageal anatomy and motility were assessed, including esophageal clearance, mucosal lesions, dilatation, and potential strictures to rule out secondary EGJOO.

#### Esophagogastroduodenoscopy (EGD)

The presence of esophagitis, esophageal dilatation, tortuosity, stasis of residual food, and resistance at the EGJ were evaluated and recorded. Los Angeles (LA) classification was used to grade esophagitis. In addition, structural pathologies such as hiatal hernia > 2 cm and strictures were assessed to exclude secondary EGJOO patients.

#### High-Resolution Impedance Manometry (HRIM)

A standard protocol was followed after placing a 4.2-mm solid-state catheter with 36 pressure sensors spaced 1 cm apart. After the calibration of sensors, baseline measurements were recorded, followed by ten standard swallows of saline, separated by at least 20 s. Tracings were analyzed using the ManoView software (Medtronic, Minneapolis, MN) to assess manometric characteristics of LES, esophageal body, and bolus clearance. EGJOO was diagnosed as an integrated relaxation pressure (IRP) > 15 mmHg with a preserved esophageal peristalsis based on Chicago classification version 3.0 criteria.^[1]^ In our practice, a suspected manometric diagnosis of EGJOO is always confirmed with additional modalities such as EndoFLIP and barium esophagogram.

#### Esophageal pH Monitoring

Patients underwent 48-h Bravo pH monitoring preoperatively and 1 year after surgery. Bravo pH capsule (Medtronic, Minneapolis, MN) was placed 5 cm above the EGJ during EGD. Patients on proton pump inhibitors (PPI) held their medications 10 days before pH testing. Abnormal distal esophageal acid exposure was defined as a DeMeester score greater than 14.7. Preoperative reflux episodes were examined to discriminate pathologic reflux from food fermentation.

### Surgical Technique

#### Peroral Endoscopic Myotomy (POEM)

All procedures were performed under general anesthesia. An anterior submucosal cushion was created by a submucosal lifting agent 2 cm above the desired proximal esophageal myotomy extent. An approximately 2 cm mucosotomy was performed at the 12 o’clock position to create a sub-mucosal tunnel using a triangle tip (TT) electrosurgical knife. Once the access was created, the endoscope was advanced into the submucosal space to clearly identify muscular layers. The tunnel was extended to the EGJ, and a further 2 cm onto the gastric cardia. Circular muscles were divided from proximal to distally by using a TT knife to preserve the longitudinal muscle layers of the esophagus and stomach. Adequate myotomy and decreased resistance at the EGJ were evaluated and confirmed endoscopically. The submucosal tunnel was then irrigated with gentamycin solution, and the mucosal incision was closed using endoscopic Resolution 360 Clips (Boston Scientific, Natick, MA).

#### Heller Myotomy and Dor Fundoplication (HMD)

HMD procedures were performed via a laparoscopic approach. Circumferential dissection of the gastroesophageal junction and lower esophagus was performed to mobilize them from the mediastinal structures and crura while both vagus nerves were preserved. An intraoperative EGD was performed to aid identification of the location of the EGJ. Long esophageal myotomy was then performed to the level of submucosa with division of longitudinal and circular muscle, extending 6 cm cephalad along the anterior esophagus from the gastroesophageal junction and 2 cm below the EGJ. The crura were then loosely approximated. The short gastric vessels were routinely divided, and an anterior 180° Dor fundoplication was constructed. Adequate myotomy and appropriate-fashioned fundoplication were confirmed endoscopically.

#### Follow-Up Protocol

All patients were admitted for inpatient hospital observation. Patients were evaluated for an esophageal leak with a water-soluble contrast esophagogram on the first postoperative day. They were then discharged on a liquid diet.

Subjective postoperative outcomes were evaluated at routine visits at 2 weeks, 6 weeks, 6 months, 1 year, and then annually after surgery. During these visits, patients were assessed for disease-specific symptoms and procedure-related complications. In addition, they were asked to complete similar preoperative questionnaires, including Eckardt symptom score and GERD-HRQL. Objective foregut evaluation was offered to patients 12 months after surgery.

### Outcomes and Definitions

Favorable outcome was defined as an Eckardt score of 3 or less at least 1 year after surgery. Objective GERD was defined as either a DeMeester score greater than 14.7 or a LA grade C or D esophagitis.

### Statistical Analysis

Values were expressed as either mean with standard deviation (SD) or median with interquartile range (IQR) for continuous variables and frequency and percentage for categorical variables. Data were analyzed using Pearson’s chi-square test for categorical variables and the Mann–Whitney *U* test for continuous variables. Type III test of fixed effect was performed for each of measures pre- and post-operative parameter using a generalized linear mixed model, in which subject effect was included in the analysis. A *p*-value < 0.05 was considered to be statistically significant. All statistical analyses were performed using the SAS software (version 9.4; SAS Institute, Cary, NC).

## Results

The final study population consisted of 52 patients with primary EGJOO who completed 1-year follow-up after surgery. All these patients had an abnormal median IRP and a complementary test confirming the diagnosis of EGJOO. Delayed transit time on timed barium esophagram was present in 25 (48.1%) patients, 13 of which also had tablet retention, and decreased distensibility index on functional lumen imaging probe (FLIP) in the remaining 27 (51.9%) patients.

Heller myotomy with Dor fundoplication was performed in 35 (67.3%) patients and POEM in 17 (32.7%) patients. Baseline demographic and clinical data for each procedure are shown in Table 1. The HMD group had a higher median (IQR) baseline total GERD-HRQL score (31 (21.5–45) vs. 16 (10–26); *p* = 0.019), with higher heartburn (12 (1–22.5) vs. 1.5 (0–10); *p* = 0.029) and regurgitation (9.5 (7–19) vs. 5 (2–8); *p* = 0.016) scores. In addition, there were 19 (65.5%) patients with an abnormal preoperative DeMeester scores in the HMD group, but no patients had preoperative objective reflux in the POEM group (*p* < 0.001). The pH monitoring tracings in all 19 (100%) patients with an abnormal DeMeester score showed no evidence of fermentation. There were 9 patients in the HMD group with a 2-cm or smaller HH, and 2 patients in the POEM group with a 1-cm HH. All preoperative HRM characteristics, including LES length and pressure, esophageal body metrics, and impedance analysis, were comparable between groups (Table 2).

**Table 1:** Comparison of baseline demographic and clinical characteristics between groups

Characteristics	HMD (*N* = 35)	POEM (*N* = 17)	*p* value
Age, median (IQR)	63 (53–70)	67 (49–80)	0.439
Sex (Female), *N* (%)	24 (68.6%)	10 (58.8%)	0.543
BMI, median (IQR)	28 (24–30)	29 (26–32)	0.135
Duration of symptoms, *y* median (IQR)	4 (2–10)	6 (4–9)	0.387
Eckardt score, median (IQR)			
Total score	7.0 (5.0–8.0)	6.0 (5.0–8.0)	1.000
Weight loss	1.0 (1.0–2.0)	1.0 (1.0–2.0)	0.337
Dysphagia	2.0 (1.0–2.0)	2.0 (2.0–3.0)	0.166
Chest pain	1.0 (0.0–2.0)	1.0 (0.0–1.0)	0.421
Regurgitation	2.0 (1.0–3.0)	2.0 (2.0–2.0)	0.761
GERD-HRQL, median (IQR)			
Total score	31.0 (21.5–45.0)	16.0 (10.0–26.0)	0.019
Heartburn score	12.0 (1.0–22.5)	1.5 (0.0–10.0)	0.029
Regurgitation score	9.5 (7.0–19.0)	5.0 (2.0–8.0)	0.016
Esophagitis, *N* (%)	5 (14.3%)	0 (0.0%)	0.159
Presence of hiatal hernia	9 (25.7%)	2 (11.7%)	0.292
DeMeester score, median (IQR)	24 (10.9–39.9)	5.8 (5.5–8.8)	0.019
Objective GERD; *N* (%)	19 (54.2%)	0 (0.0%)	0.001

**Table 2:** Comparison of baseline objective characteristics between groups

Characteristics	HMD (*N* = 35)	POEM (*N* = 17)	*p* value
LES length, median (IQR)	3.5 (2.7–4.0)	3.0 (2.9–4.0)	0.968
LES intraabdominal length, median (IQR)	1.8 (1.1–2.2)	2.2 (0.9–2.5)	0.463
LES resting pressure, median (IQR)	48.2 (33.9–58.8)	43.0 (30.0–67.6)	0.792
Integrated relaxation pressure, median (IQR)	22.4 (17.2–28.2)	23.0 (18.1–32.6)	0.283
Distal contractile integral, median (IQR)	2153 (1219–4666)	1920 (1606–5459)	0.906
Mean wave amplitude, median (IQR)	106 (67–150)	85 (60–124)	0.277
Percent intact swallows, median (IQR)	80.0 (60.0–100.0)	60.0 (30.0–100.0)	0.350
Percent failed swallows, median (IQR)	10.0 (0.0–40.0)	0.0 (0.0–30.0)	0.966
Percent ineffective swallow, median (IQR)	10.0 (0.0–40.0)	10.0 (0.0–70.0)	0.837
Percent peristalsis, Median (IQR)	80.0 (40–100.0)	50.0 (30.0–70.0)	0.120
Incomplete bolus clearance, median (IQR)	70.0 (10.0–90.0)	80.0 (50.0–90.0)	0.071

The HMD group had longer median (IQR) operation time (210 (160–249) vs. 117 (108–172); *p* = 0.005) and hospital stay (2 (1–2) vs. 1 (1–1); *p* < 0.001). Total (8 (7–8) vs. 13 (10–17); *p* < 0.001) and esophageal (5 (5–6) vs. 11 (7–15); *p* = 0.001) myotomy lengths were shorter in the HMD group, but the gastric myotomy length was similar between surgery groups (*p* = 0.078) (Table 3).

**Table 3:** Comparison of perioperative factors between groups

Characteristics	HMD (*N* = 35)	POEM (*N* = 17)	*p* value
Myotomy length, cm			
Total	8.0 (7.0–8.0)	13.0 (10.0–17.0)	< 0.001
Esophageal	5.0 (5.0–6.0)	11.0 (7.0–15.0)	0.001
Gastric	2.5 (2.0–3.0)	2.0 (2.0–3.0)	0.078
Operation time, min, median (IQR)	210 (160–249)	117 (108–172)	0.005
Hospital stay, day, median (IQR)	2 (1–2)	1 (1–1)	< 0.001
Perioperative complications	0 (0%)	1 (5.9%)	0.327
90-day readmission, *N* (%)	1 (2.9%)	1 (5.9%)	1.000

### Surgical Outcome After Heller Myotomy with Dor Fundoplication

**Fig. 1:**
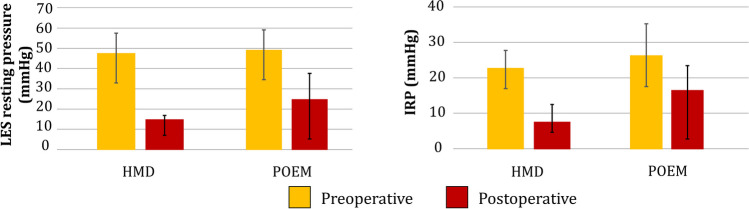
Comparison of pre and post-operative LES characteristics in both HMD and POEM. For HMD, there was a decrease in IRP (*p* < 0.001) and LES resting pressure (*p* < 0.001). For POEM, there was a decrease in IRP (*p* = 0.048) and LES resting pressure (*p* = 0.042)

**Fig. 2:**
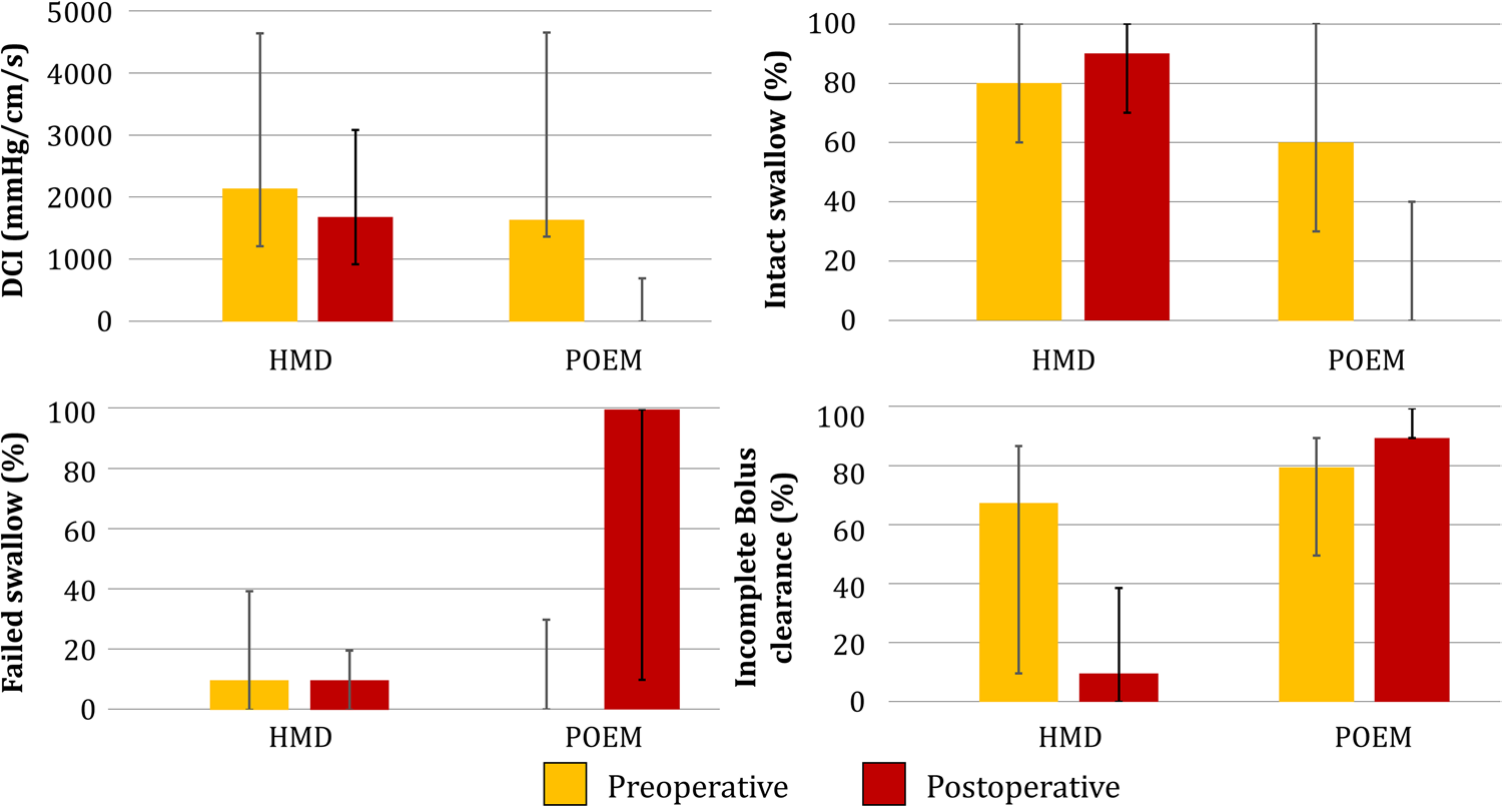
Comparison of pre- and post-operative esophageal body characteristics for HMD and POEM. After HMD, DCI (*p* = 0.118), percent intact swallow (*p* = 0.355), and percent failed swallow (*p* = 0.223) remained unchanged, whereas percent incomplete bolus clearance significantly decreased (*p* = 0.010). After POEM, there was a significant decrease in DCI (*p* = 0.035) and percent intact swallow (*p* = 0.049) and a significant increase in percent failed swallows (*p* = 0.032), whereas percent incomplete bolus clearance remained unchanged (*p* = 0.539)

At a median (IQR) of 21.1 (12–35) months, a favorable outcome was achieved in 30 (85.7%). Eckardt scores decreased from 7.0 (5–8) to 1.0 (0–2) (*p* < 0.001) (Table 4). GERD-HRQL total score decreased from 31.0 (22–45) to 4.0 (0–19) (*p* < 0.001) with heartburn and regurgitation total scores also showing significant improvement (*p* values: < 0.001 and 0.018, respectively). The rate of objective reflux decreased from 54.2 to 25.9% (*p* = 0.033). On manometry, there was a significant decrease in the LES resting pressure from 48.2 (34–59) to 12.7 (8–17) (*p* < 0.001) and IRP from 22.4 (17–28) to 7.9 (3–11) (*p* < 0.001) (Fig. 1). There were no changes in esophageal body characteristics; however, incomplete bolus clearance improved from 70% (10–90) to 10% (0–40) (*p* = 0.010) (Fig. 2).

**Table 4:** Comparison of preoperative and postoperative clinical and objective characteristics

	Heller myotomy with dor fundoplication			POEM		
Characteristics	Preop (*N* = 35)	Postop (*N* = 35)	*p* value	Preop (*N* = 17)	Postop (*N* = 17)	*p* value
Eckardt score, median (IQR)						
Total score	7.0 (5–8)	1.0 (0–2)	< 0.001	6.0 (5–8)	1.0 (1–2)	< 0.001
Weight loss	1.0 (1–2)	0.0 (0–0)	< 0.001	1.0 (1–2)	0.0 (0–0)	0.001
Dysphagia	2.0 (1–2)	0.0 (0–1)	< 0.001	2.0 (2–3)	1.0 (0–1)	< 0.001
Chest pain	1.0 (0–2)	0.0 (0–1)	< 0.001	1.0 (0–1)	0.0 (0–1)	0.038
Regurgitation	2.0 (1–3)	0.0 (0–0)	< 0.001	2.0 (2–2)	0.0 (0–0)	< 0.001
GERD-HRQL, median (IQR)						
Total score	31.0 (22–45)	4.0 (0–19)	< 0.001	16.5 (10–26)	11.0 (2–18)	0.854
Heartburn	12.0 (1–23)	0.0 (0–8)	< 0.001	1.5 (0–10)	2.0 (0–10)	0.408
Regurgitation	9.5 (7–19)	1.0 (0–6)	0.018	5.0 (2–8)	1.0 (0–7)	0.662
Esophagitis LA C/D, *N* (%)	1 (2.9%)	6 (20.7%)	0.060	0 (0.0%)	1 (7.1%)	0.451
DeMeester score, median (IQR)	24 (11–40)	8.7 (1–32)	0.724	5.8 (6–9)	54.3 (29–62)	0.134
Objective GERD *N* (%)	19 (54.2%)	7 (25.9%)	0.033	0 (0.0%)	8 (61.5%)	< 0.001

### Surgical Outcome after POEM

At a median (IQR) of 22.6 (13–38) months, favorable outcome was achieved in 14 (82.4%) after POEM (*p* = 0.753). Eckardt scores decreased from 6.0 (5–8) to 1.0 (1–2) (*p* < 0.001) (Table 4). There was no change in the GERD-HRQL total score (*p* = 0.854) or heartburn (0.408) or regurgitation (0.662) scores. There was a significant increase in the rate of objective reflux (0 to 62%; *p* < 0.001). Of the two patients with a 1-cm preoperative hiatal hernia, one developed an abnormal postoperative DeMeester score, and the other had no objective reflux. On manometry, there was a significant decrease in the LES resting pressure from 43 (30–68) to 31.2 (5–34) (*p* = 0.042) and IRP (23.0 (18–33) to 12.4 (10–32); *p* = 0.048) (Fig. 1). For esophageal body characteristics, there was a significant decrease in DCI (1920 (1600–5500) to 0 (0–814); *p* = 0.035) and percent intact swallows (60% (30–100) to 0% (0–40); *p* = 0.049), and an increase in failed swallows (0% (0–30) to 100% (10–100); *p* = 0.032). Percent bolus clearance did not improve (*p* = 0.539) (Fig. 2).

### Comparison Heller Myotomy with Dor Fundoplication and POEM Outcomes

Outcomes are compared between HMD and POEM in Table 5. There was no difference in favorable outcome, postoperative Eckardt score, or GERD-HRQL score. However, the POEM group had a significantly higher rate of PPI use (53% vs. 23%; *p* = 0.030) and objective reflux (61.5% vs. 25.9%; *p* = 0.041).

**Table 5:** Comparison of postoperative outcomes between groups at 1 year

Characteristics	HMD (*N* = 35)	POEM (*N* = 17)	*p* value
Favorable outcome; *N* (%)	30 (85.7%)	14 (82.4%)	0.753
Eckardt score, median (IQR)			
Total score	1.0 (0.0–2.0)	1.0 (1.0–2.0)	0.424
Weight loss	0.0 (0.0–0.0)	0.0 (0.0–0.0)	0.651
Dysphagia	0.0 (0.0–1.0)	1.0 (0.0–1.0)	0.237
Chest pain	0.0 (0.0–1.0)	0.0 (0.0–1.0)	0.701
Regurgitation	0.0 (0.0–0.0)	0.0 (0.0–0.0)	0.732
Need for additional procedure	2 (5.7%)	1 (5.8%)	0.980
GERD-HRQL, median (IQR)			
Total score	4.0 (0.0–19.0)	11.0 (2.0–18.0)	0.370
Heartburn	0.0 (0.0–8.0)	2.0 (0.0–10.0)	0.269
Regurgitation	1.0 (0.0–6.0)	1.0 (0.0–7.0)	0.700
Patient satisfaction; *N* (%)	27 (87.1%)	10 (66.7%)	0.127
PPI use; *N* (%)	8 (23%)	9 (53%)	0.030
Objective GERD; *N* (%)	7 (25.9%)	8 (61.5%)	0.041
DeMeester score, median (IQR)	8.7 (1.1–31.9)	54.3 (28.7–61.7)	0.056

Postoperative manometric characteristics are compared in Table 6. Postoperative median LES resting pressure (*p* = 0.290) and IRP (*p* = 0.341) were similar between groups. POEM group had significantly higher rates of median (IQR) ineffective swallows (100 (10–100) vs. 10 (0–20); *p* = 0.006) and incomplete bolus clearance (90 (90–100) vs. 10 (0–40); *p* = 0.004), and lower median percent peristalsis (10 (0–80) vs. 90 (60–100); *p* = 0.014), median DCI (0 (0–814) vs. 1695 (929–3101); *p* = 0.004), and mean wave amplitude (26.8 (11.8–33) vs. 79.9 (66.3–130.2); *p* = 0.003).

**Table 6:** Comparison of postoperative objective characteristics between groups

Characteristics	HMD	POEM	*p* value
LES length, median (IQR)	2.9 (2.1–4.0)	2.6 (2.2–2.9)	0.415
LES intraabdominal length, median (IQR)	1.5 (0.4–2.5)	2.0 (1.0–2.9)	0.305
LES resting pressure, median (IQR)	12.7 (7.8–16.9)	31.2 (5.2–33.6)	0.290
Integrated relaxation pressure, median (IQR)	7.9 (2.9–10.7)	12.4 (10.4–31.9)	0.091
Distal contractile integral, median (IQR)	1695 (929–3101)	0 (0–814)	0.004
Mean wave amplitude, median (IQR)	79.9 (66.3–130.2)	26.8 (11.8–33.0)	0.003
Percent intact swallows, Median (IQR)	90.0 (70.0–100.0)	0.0 (0.0–40.0)	0.006
Percent failed swallows, median (IQR)	10.0 (0.0–20.0)	100.0 (10.0–100.0)	0.018
Percent ineffective swallow, median (IQR)	10.0 (0.0–20.0)	100.0 (60.0–100.0)	0.006
Percent peristalsis, median (IQR)	90.0 (60.0–100)	10.0 (0.0–80.0)	0.014
Incomplete bolus clearance, median (IQR)	10.0 (0.0–40.0)	90.0 (90.0–100.0)	0.004

## Discussion

The underlying pathophysiology of EGJOO is failure of lower esophageal sphincter (LES) relaxation. This pathophysiology is shared by other disorders of EGJ outflow, such as achalasia. As such, the management EGJOO often mirrors that of achalasia. The goal of surgical intervention for EGJOO is to alleviate dysphagia by directly addressing the underlying pathophysiology through myotomy. Surgeons have been performing Heller myotomy to achieve this goal in achalasia patients for more than 100 years and have been using a laparoscopic approach since the early 90 s. Then, in 2010, the POEM technique was introduced by Inoue et al.^[10]^. The appeal of an entirely endoscopic alternative to myotomy has led to rapid and widespread adoption, with studies showing a 19-fold increase in its use over an 8-year period.^[11]^ Although the surgical management of primary EGJOO is uncommon, studies have demonstrated that both laparoscopic and endoscopic myotomy are independently capable of attacking obstructive pathophysiology and relieving symptoms of primary EGJOO.^[12]^ However, despite following a similar treatment paradigm as achalasia, the distinguishing characteristic of EGJOO is that the esophageal body is preserved, a factor that previous studies have not assessed postoperatively. In this study, we assessed both HMD and POEM for the treatment of primary EGJOO and found that both procedures are equally effective at alleviating dysphagia symptoms. However, POEM comes at the cost of the function of esophageal body physiology, resulting in a failure to improve bolus clearance, and a high rate of postoperative GERD.

We found that HMD and POEM were highly and comparably effective at palliating dysphagia, consistent with the findings of the limited studies on the impact of each surgery on primary EGJOO. A prospective single-center study of 15 patients with EGJOO who underwent POEM reported a clinical success rate of 93% after surgery.^[13]^ Similarly, a multicenter retrospective study reported a 94% clinical success rate after POEM. Studies of HMD for primary EGJOO primarily consist of case series, but have shown similarly successful outcomes. Scherer et al. analyzed 1000 consecutive HRM, diagnosed 16 patients with primary EGJOO, of which 3 underwent Heller myotomy with resolution of symptoms in all patients.^[14]^ This study highlights the feasibility of surgical myotomy for primary EGJOO, and also the frequency with which these patients are diagnosed and undergo surgery. Salvador et al. reported a relatively large series of 25 patients who underwent laparoscopic HMD for primary EGJOO and found 96% favorable outcome. They also compared this favorable outcome rate to HMD for achalasia type 1 (96%), type 2 (98.7%), and type 3 (96.2%), and found that they were equivalent.^[7]^ These studies suggest that both laparoscopic and endoscopic esophageal myotomy are highly effective for palliation of dysphagia in multiple disorders of EGJ outflow.

Both procedures resulted in a similar degree of relief at the EGJ with no significant difference in the postoperative IRP. However, we found that postoperative IRP was higher in the POEM group. This finding has been previously described in the literature. A systematic review and meta-analysis comparing HMD (5834 patients) and POEM (1958 patients) similarly found that post-POEM IRP was higher than post-HMD IRP.^[9]^ A series of 500 POEMs reported a mean (SD) IRP 12.2 (6.0) at 1- to 2-year follow-up, consistent with our post-POEM IRP.^[15]^ Similarly, a retrospective study with 32 patients who underwent POEM reported a mean postoperative IRP of 10.6.^[16]^ An important difference between HMD and POEM is that the longitudinal muscle layer was preserved in POEM. Neuroanatomical studies have found that the longitudinal muscle layer contributes to IRP. A study using 3-D high resolution manometry to study the individual contributions of muscle layers before during and after bolus transit found that the longitudinal muscle layer contributes to the EGJ high-pressure zone.^[17]^ This finding suggests that preservation of the longitudinal muscle layer may preserve its contribution to residual EGJ pressure, which was reflected in the marginally higher IRP in POEM compared to HMD.

The defining characteristic that sets EGJOO apart from other motility disorders of EGJ outflow is that esophageal body function is largely preserved. As myotomy is most commonly used to treat esophageal motility disorders characterized by complete failure of peristalsis, little attention has been paid to the impact of myotomy on esophageal body function. However, we found that POEM results in near complete elimination of esophageal body function, transforming patients with no failed swallows to 100% failed swallows with a median DCI of zero, while incomplete bolus clearance failed to improve. This loss of esophageal body function is a novel finding in the literature of POEM for EGJOO. However, studies of POEM for jackhammer esophagus, a motility disorder characterized by high-amplitude disordered esophageal body contractility, have found similar results. Bechara et al. found that the median (IQR) DCI in four patient who underwent POEM for Jackhammer esophagus decreased from 16,860 mmHg·sec·cm (14,000–33,000) to 186 mmHg·sec·cm (111–1127), with 75% meeting criteria for ineffective esophageal motility.^[18]^ These findings suggest that POEM results in severe loss of esophageal body function, effectively transforming the esophagus from a dynamic organ with complex physiology to a mere tube connecting the mouth to the stomach. In achalasia, this effect is likely masked by baseline aperistalsis. By contrast, HMD had no deleterious impact on esophageal body function, and even significantly improved bolus clearance to only 10% incomplete.

A proposed advantage of the POEM procedure is the ability to tailor the length of myotomy to the patient’s diagnosis and extent of dysfunctional esophagus. However, there is no well-established esophageal myotomy length for primary EGJOO. Salvador et al. reported their HMD technique with a 7–8-cm-long anterior esophageal myotomy extending to 1.5–2 cm to proximal stomach, similar to our 8 cm myotomy extending 2.5 cm on to the cardia.^[7]^ By contrast, endoscopic surgeons perform a substantially longer myotomy with POEM. A retrospective multicenter study of 55 patients who underwent POEM for primary EGJOO reported a mean total myotomy length of 12.58 cm, similar to our median length of 13 cm.^[6]^ We found that esophageal myotomy length was significantly longer in patients who underwent POEM compared to HMD. This difference is the likely reason that esophageal body characteristics remained relatively stable, with bolus clearance improving after HMD, while POEM resulted in near complete loss of esophageal body function. The tailored length may enable surgeons to address spasm that extends to the mid-esophagus. However, a longer myotomy of the esophageal body may induce aperistalsis. Studies using a combination of mathematical modeling and assessment of the neurophysiologic innervation of the muscle layers of the esophagus have demonstrated the majority of peristaltic activity is generated by inhibitory innervation of the inner circular layer.^[19]^ The outer longitudinal muscle layer’s role in peristalsis is to augment and stabilize the activity of the inner circular layer.^[20]^ Additionally, neuroanatomical studies have demonstrated that these inhibitory fibers that innervate the inner circular layer lie between the muscle layers, putting them at risk of damage during a circular myotomy.^[21]^ It is likely that the tailored myotomy length in POEM both the circular layer and its innervation, resulting in the pronounced loss of function. The resultant aperistalsis can also worsen esophageal acid clearance and increase objective reflux. Consequently, patients who underwent POEM had a substantial improvement in Eckardt score but had no improvement in the GERD-HRQL regurgitation or heartburn scores.

The mechanism by which myotomy alleviates dysphagia is the reduction of resistance at the EGJ. The goal is to improve outflow, but when resistance decreases too much it allows backflow, resulting in reflux. Therefore, GERD is a common complication following myotomy, particularly without the addition of an antireflux procedure. Limited studies of patients who underwent HMD for EGJOO have found relatively low rates of postoperative abnormal acid exposure at 14.3%.^[7]^ A retrospective comparative study of 35 patients who underwent myotomy without fundoplication and 133 who underwent laparoscopic HMD for achalasia found that the addition of the Dor fundoplication decreased the rate of postoperative pathologic reflux from 60 to 17%.^[22]^ This study highlights the high risk of GERD after myotomy and the mitigating impact of a post-myotomy antireflux surgery. Similarly, we found that despite a comparatively high-degree of preoperative reflux, HMD resulted in significant improvement in distal esophageal acid exposure. By contrast, POEM does not routinely employ an antireflux procedure, resulting in high rates of GERD. A prospective single-center trial of 15 patients who underwent POEM for EGJOO reported that 7 out of 10 patients had abnormal DeMeester scores one year after surgery.^[13]^ A recent study from our center of 183 patients who underwent POEM found that 50.5% of patients developed postoperative GERD, with 19.2% developing LA grade D esophagitis or a DeMeester > 50. Moreover, there was no difference in degree of postoperative acid exposure between patients who underwent POEM for EGJOO, jackhammer esophagus, or achalasia subtypes.^[23]^ Likewise, a retrospective multicenter study with a large cohort of 55 EGJOO patients reported post-POEM abnormal acid exposure in 66% of patients.^[6]^ These studies are consistent with our finding that despite a normal baseline DeMeester score in all patients, POEM results in a significant increase in postoperative GERD. Furthermore, post-POEM GERD was significantly worse than post-HMD GERD, despite higher baseline reflux before HMD. Additionally, there was no improvement in reflux symptoms after POEM. Therefore, patients who undergo POEM should be counselled that GERD is a likely outcome without the addition of an antireflux procedure, and should be monitored accordingly. However, considering the deleterious impact on the esophageal body, postoperative GERD is another reason to avoid POEM in patients with EGJOO.

It has been suggested that the length of myotomy may influence the rate of GERD after POEM. However, in our recent study on predictors of GERD after POEM, we found that myotomy length had no impact on the rate of GERD.^[23]^ Additionally, a randomized controlled trial of short versus long myotomy found a post-POEM GERD rate of 50.9%, but no difference between myotomy length groups (*p* = 0.431).^[24]^ Therefore, while tailored myotomy length may have played a role in post-POEM peristalsis, post-POEM GERD likely developed due to the lack of fundoplication, not myotomy length.

Since the introduction of the Chicago Classification version 3.0 (CCv3) in 2015, there has been a substantial rise in both diagnosis and LES directed therapy for EGJOO. In fact, studies have showed that nearly 10% of all patients who underwent HRM met CCv3 criteria for EGJOO, with nearly one-third of these being clinically irrelevant.^[25]^ In the same period, there has been a 19-fold increase in use of POEM, while the use of HMD has remained stable.^[11]^ As a result, there has been increased concern for unnecessary treatment of benign manometrically defined EGJOO. Therefore, in the recently updated Chicago Classification version 4.0, the definition of EGJOO was changed to be more stringent and clinically relevant.^[26]^ A manometric diagnosis of EGJOO is now always considered inconclusive without supporting evidence in the form of clinically relevant symptoms (dysphagia and/or non-cardiac chest pain) and corroborating objective evidence on another esophageal physiology modality. The time period of our study precedes the publication of the CCv4 update, but at our center, a manometric diagnosis of EGJOO has always been confirmed with additional modalities such as FLIP or barium esophagogram prior to surgery. In addition, all of our patients had symptomatic EGJOO for a median of 6 years prior to surgery. Therefore, we do not believe that this study’s findings would be substantially different under CCv4 criteria.

We acknowledge certain limitations of our study design including its retrospective nature, small sample size, and non-randomization of the surgical procedures. It has been our practice to preferentially recommend HMD to patients with preoperative evidence of objective reflux. These baseline physiological differences may have had some impact on outcome. However, given our findings and available literature with regard to GERD after POEM, we would not recommend randomizing patients with preoperative reflux into a POEM arm of a study.

## Conclusion

The results of surgical myotomy are best assessed across three spectrums: dysphagia, reflux, and esophageal motility. In this study, we compared the impact Heller myotomy with Dor fundoplication and per oral endoscopy myotomy for the management of primary esophagogastric junctional outflow obstruction and found that both HMD and POEM are equivalently and highly effective at relieving dysphagia. However, POEM resulted in significantly worse objective reflux and near-complete loss of esophageal contractility and bolus clearance. Therefore, given that POEM comes at the cost of antireflux barrier and esophageal body function with comparable dysphagia symptom improvement, HMD is the procedure of choice in our center for patients considering surgery for primary EGJOO.
